# Melanocortin-4 Receptor Gene Mutations in a Group of Turkish Obese Children and Adolescents

**DOI:** 10.4274/jcrpe.4225

**Published:** 2017-09-01

**Authors:** Selma Tunç, Korcan Demir, Fatma Ajlan Tükün, Cihan Topal, Filiz Hazan, Burcu Sağlam, Özlem Nalbantoğlu, Melek Yıldız, Behzat Özkan

**Affiliations:** 1 Dr. Behçet Uz Children’s Hospital, Clinic of Pediatric Endocrinology, İzmir, Turkey; 2 Dokuz Eylül University Faculty of Medicine, Department of Pediatric Endocrinology, İzmir, Turkey; 3 Ankara University Faculty of Medicine, Department of Medical Genetics, Ankara, Turkey; 4 Dr. Behçet Uz Children’s Hospital, Clinic of Pediatrics, İzmir, Turkey; 5 Dr. Behçet Uz Children’s Hospital, Clinic of Medical Genetics, İzmir, Turkey; 6 Düzen Laboratory, Division of Genetic Diagnosis Center, Ankara, Turkey

**Keywords:** Melanocortin-4 receptor, obesity, mutation

## Abstract

**Objective:**

Melanocortin-4 receptor (MC4R) mutations are the most common known cause of monogenic obesity. Data regarding MC4R mutations in Turkish subjects are limited. To determine the prevalence of MC4R mutations in a group of Turkish morbid obese children and adolescents.

**Methods:**

MC4R was sequenced in 47 consecutive morbidly obese children and adolescents (28 girls and 19 boys, aged 1-18 years) who presented during a one-year period. Inclusion criterion was a body mass index (BMI) ≥120% of the 95th percentile or ≥35 kg/m2. Patients with chronic diseases, Cushing syndrome, hypothyroidism, or suspected syndromes that could cause obesity were excluded. Onset of obesity was before age 10 years in all subjects.

**Results:**

Mean age was 13.2±4.1 years, age at onset of obesity 5.1±2.1 years, height standard deviation (SD) score 1.21±0.93, BMI 40.0±8.8 kg/m^2^, and BMI SD score was 2.72±0.37. One novel (c.870delG) and two previously reported (c.496 G>A, c.346_347delAG) mutations were found in four (8.5%) obese children and adolescents. The novel mutation (c.870delG) was predicted to be a disease-causing frame-shift mutation using in silico analyses. Fasting glucose and lipid levels of the patients with MC4R mutation were normal, but insulin resistance was present in two of the subjects. Six more individuals with MC4R mutation (1 child, 5 adults) were detected following analyses of the family members of affected children.

**Conclusion:**

MC4R mutations are frequently found in morbid obese Turkish children and adolescents.

**What is already known on this topic?**

Melanocortin-4 receptor (MC4R) mutations are the most common known cause of monogenic obesity. Prevalence of MC4R mutations in children with severe obesity varies from 0.3% up to 6.3%, but there is no relevant published data on Turkish subjects.

**What this study adds?**

The present study reports a novel mutation and suggests that MC4R mutations are more frequent in Turkish children and adolescents with severe obesity as compared to the existing literature.

## INTRODUCTION

Genetic background in obesity is frequently polygenic and rarely monogenic (1). Among the monogenic types of non-syndromic obesity, melanocortin-4 receptor (MC4R) deficiency is presumably the most frequent and the best understood form (2). MC4R encodes the 322-amino acid 7-transmembrane G-protein-linked receptor (3). This receptor is expressed in many neurons in several areas of brain including hypothalamus and contributes to appetite regulation. Activation of MC4R by alpha-melano-stimulating hormone, which is produced following interaction of leptin with its receptor, stimulates the anorexigenic pathways and increases energy expenditure (4).

MC4R mutations result in hyperphagia, early-onset obesity, increased linear growth in childhood, increased body fat and fat-free mass, increased bone mineral density, and hyperinsulinemia (5). To date, over 150 different mutations have been reported in MC4R (4). Prevalence of MC4R mutations in children with severe obesity varies from 0.3% up to 6.3% (6,7). However, there is no such data from Turkey. The single study of MC4R in obese Turkish children was on evaluation of two polymorphisms (8).

Assessment of MC4R mutations would further be of benefit regarding treatment. Recently, setmelanotide, a MC4R agonist, was shown to be effective in treatment of patients with proopiomelanocortin deficiency (9). It might also be effective in treatment of MC4R deficiency.

The aim of this study was to establish the prevalence of MC4R mutations in a group of Turkish children and adolescents with morbid obesity.

## METHODS

The study was conducted in one of the major tertiary children’s hospitals in the region. Consecutive subjects with morbid obesity were recruited from the pediatric endocrinology clinic during a 1-year period. Morbid obesity was defined as body mass index (BMI) ≥120 percent of the 95^th^ percentile values or a BMI ≥35 kg/m^2^ (whichever is lower). This corresponds to approximately the ≥99^th^ percentile or BMI standard deviation (SD) score ≥2.33 (10). Cases with chronic diseases (cardiovascular, gastrointestinal, and respiratory), a history of drug use (steroids and antipsychotics), endocrine pathology resulting in secondary obesity, or suspected syndromes associated with obesity (including Prader-Willi and Laurence-Moon-Biedl syndromes) were excluded. Following written informed consent from their legal representatives consistent with the Helsinki declaration, 47 unrelated Turkish morbid obese children and adolescents of ages 1-18 years (28 girls and 19 boys) were included in the study. Onset of obesity was before the 10^th^ year of life in all subjects.

Height was measured to the nearest 0.5 cm. Body weight (barefoot, wearing light clothes) was measured using an electronic scale sensitive to the nearest 100 g. Body weight, height, and BMI were recorded, and their SD scores were calculated using Turkish national anthropometric references (11).

All subjects underwent a clinical examination and blood samples were obtained after 12-h fasting for biochemical parameters including glucose, insulin, triglycerides, total cholesterol, high density lipoprotein cholesterol, and low density lipoprotein cholesterol; genetic analyses were performed. The study was approved by the institutional ethics committee (2015/17-01).

### Genetic Analyses

Peripheral blood samples were collected in EDTA tubes. Genomic DNA was extracted from blood lymphocytes by standard procedures. All exons and adjacent intronic regions of MC4R were amplified by polymerase chain reaction (PCR) using previously reported primer pairs (12). The products of PCR were purified and directly sequenced using the Big Dye Sequencing kit (Applied Biosystems, Foster City, CA, USA) on an ABI 3100 automated DNA sequencer (Applied Biosystems, Foster City, CA, USA). DNA sequences were analyzed using the SeqScape Software version 2.5 and Sequencing Analysis Software version 5.1 for the identification of mutations. Genetic analyses were also made in the parents and siblings of the index cases.

### Statistical Analysis

The data were statistically analyzed using SPSS 15.0 (Chicago, IL, USA). Mann-Whitney U-test and chi-square test were used to compare numerical and categorical variables, respectively. A p-value of <0.05 was chosen to represent statistical significance. Data were presented as mean ± SD or n (%).

## RESULTS

The study included 47 morbid obese children and adolescents (28 girls and 19 boys, aged 1-18 years). Mean age was 13.2±4.1 years, mean age at onset of obesity 5.1±2.1 years, mean height SD score 1.21±0.93, mean BMI 40.0±8.8 kg/m^2^, and BMI SD score was 2.72±0.37. Comparison of mutation carriers and non-carriers regarding anthropometric (BMI SD score, height SD score, weight SD score) and biochemical (fasting blood glucose, lipids, insulin, free thyroxine, thyroid-stimulating hormone, adrenocorticotropic hormone, and cortisol) variables revealed no statistically significant differences except for age at onset of obesity (Table 1).

We detected 3 distinct variants of MC4R (c.870delG, c.496 G>A, c.346_347delAG) in four patients (8.5%). The c.870delG mutation was novel; the remaining mutations have been reported previously (4). The families with a MC4R mutation are presented below in chronological order of diagnosis and evaluation of cases. Genotypes and phenotypic characteristics of the index cases are summarized in Table 2.

### Family 1

**Figure 1:**
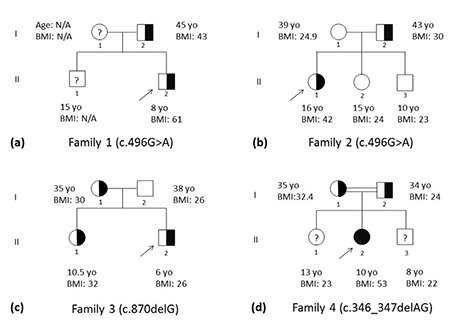
Pedigrees of the four families with melanocortin-4 receptor mutations BMI: body mass index, N/A: non-available, yo: year-old, arrows indicate the index cases, and question marks indicate unknown mutation status

An 8-year-old boy (Patient 1-II-2) who suffered from obesity since age 3 years was the first index case. He was born at term (3800 g) following an eventless pregnancy. Motor and mental developmental stages were normal. Hyperphagia (demanding more food immediately after a meal) was present. His parents were not relatives. His father was obese since childhood. The height of the index case was 148 cm (SD score 3.4), weight 134 kg (SD score 4.27), BMI 61 kg/m^2^ (SD score 3.05). Physical examination revealed acanthosis nigricans. Fasting insulin and glucose levels were 29.8 mIU/L and 90 mg/dL, respectively. MC4R analysis revealed a previously reported heterozygous c.496G>A (p.V166I) mutation. A family segregation analysis for this mutation showed that his father (Patient 1-I-2, BMI 43 kg/m^2^) had the same mutation as well (Figure 1a).

### Family 2

The second index case was a 16-year-old female (Patient 2-II-3) who was known to be obese since age 4 years. She was born at term (3600 g) following an eventless pregnancy. She had attained normal motor and mental developmental stages. Hyperphagia was not reported. Her parents were not consanguineous. The father was slightly obese (BMI 30 kg/m^2^). Her height was 171.3 cm (SD score 1.36), weight 122 kg (SD score 2.47), BMI 42 kg/m^2^ (SD score 2.47). Physical examination revealed no other findings. MC4R analysis revealed the same mutation as in Family 1: heterozygous c.496G>A (p.V166I) (Figure 1b). Family 1 and Family 2 were not related. Among the family members, only the father was carrying the mutation.

### Family 3

**Figure 2:**
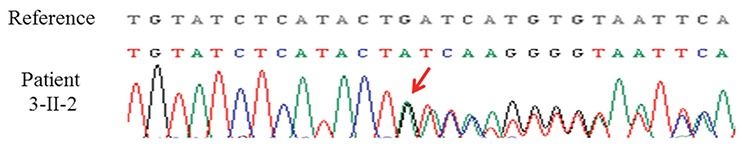
Heterozygous deletion of guanine (arrow) at nucleotide 870 results in a frame-shift mutation

A novel mutation (heterozygous c.870delG, Figure 2) was detected in a 6-year-old boy who was reported to be obese since the age of 2 years (Patient 3-II-2). He was born at term (3900 g) following a normal pregnancy. His motor and mental developmental stages were normal. Hyperphagia was reported to be present. His parents were not relatives. His mother was obese since childhood. The height of the index case was 126 cm (SD score 1.94), weight 41 kg (SD score 3.41), BMI 26 kg/m^2^ (SD score 3.01). Remaining physical examination was normal. The novel MC4R mutation was predicted to be a disease-causing frame-shift mutation (p.I291SfsX10) using in silico analyses. Results of bioinformatics analyses of the mutation with PolyPhen2 and Mutation Taster were in agreement: probably damaging (score, 0.999) and disease-causing (probability, 1.000), respectively. His mother (BMI 30 kg/m^2^) and sister (10.5 years, BMI 32 kg/m^2^, SD score 2.3) were found to have the same mutation (Figure 1c).

### Family 4

A previously reported c.346_347delAG (p.S116Ffsx6) mutation was found in homozygous state in a 10-year-old female (Patient 4-II-2) with consanguineous parents. She was obese since 1 year of age. She was born by cesarean section at term (4200 g). Her motor and mental developmental stages were normal. Hyperphagia was described. BMI values of her mother and father were 32.4 kg/m^2^ and 24 kg/m^2^, respectively. Her height was 147 cm (SD score 1.06), weight 114 kg (SD score 3.87), BMI 53 kg/m^2^ (SD score 3.01). Physical examination revealed acanthosis nigricans. Fasting insulin and glucose levels were 28 mIU/L and 84 mg/dL, respectively. Both parents were heterozygous for the mutation (Figure 1d).

## DISCUSSION

To the best of our knowledge, this is the first published study to assess MC4R mutations in Turkish children and adolescents with morbid obesity. We found three different MC4R mutations in four of 47 subjects (8.5%). Screening of family members revealed more affected cases.

Until now, a variable frequency of MC4R mutations (0.3-6.3%) was reported in obese children. This wide range apparently seems to be due to different inclusion criteria and ethnic background in relevant studies. Wang et al (13) included non-syndromic Chinese children with a BMI >97^th^ percentile (nearly 2 SD score) and found that 1.5% of the cases were carrying a MC4R mutation. Santoro et al (14) included tall (>2 SD score) and severely obese (BMI >3 SD score) Italian children who started to gain weight before 10 years of age and with at least one obese parent. They have found three mutations in five obese children (1.6%) (14). Interestingly, frequency of MC4R mutations was only 0.95% among 210 Slovak children whose mean BMI SD score was 4.86±1.7 (7). However, Dubern et al (15) included 63 severely obese (BMI>SD score) French children with non-syndromic and early-onset obesity and found a higher prevalence: 6.3%. In the present study, we found an even higher rate of MC4R mutations compared to the existing literature. This might be due to inclusion of cases who had more severe obesity (approximately ≥99^th^ percentile or BMI SD score ≥2.33) which started early in life. Of note, in an unpublished study from another center in our city, frequency of MC4R mutations was reported to be 8.6% among 93 obese children and adolescents (mean age 7.3±3.7 years) who started to gain weight before 6 years of age and had a history of early-onset obesity in a first-degree relative (16).

There is only one study assessing MC4R mutations in morbidly obese Turkish adults. Mergen et al (17) included 40 subjects with onset of severe obesity before 10 years of age and a history of obesity in at least one family member. There was only one affected case (BMI 41.7 kg/m^2^) with a p.N247S mutation. They reported a lower mutation rate (2.5%) despite having the same ethnic background. However, we cannot make a comparison since definition of severe obesity and BMI values of the study group were not provided (17). Furthermore, it is known that some of the MC4R mutation carriers are obese during childhood but not in adulthood (7,14,18).

We did not detect any differences in the anthropometric and biochemical variables between mutation carriers and non-carriers (Table 1). However, the age of onset of obesity was significantly lower in mutation carriers compared to non-carriers. These findings were similar to those of other studies (7,13,14,19). In addition, hyperphagia, tall stature, and hyperinsulinemia were not present in all affected cases. Farooqi et al (20) reported that only some of MC4R mutation carriers had hyperinsulinemia.

One of the mutations (c.870delG) we detected was not reported previously. This novel mutation was present only in the affected cases in Family 3 and it was predicted to be a disease-causing frame-shift mutation using in silico analyses. In case 4-II-2, a homozygous MC4R mutation was detected. It was reported that age at onset of obesity was earlier and obesity was more severe in homozygous mutation carriers compared to heterozygous mutation carriers (5,20). In our case, obesity began at an earlier age, but BMI SD score was not higher. In addition, while both parents were heterozygous for the mutation, only the mother was obese. Several studies have also reported that mutation carriers would have a normal BMI value (7,14,18). According to Dubern et al (1), the phenotypic difference between the parents may be caused due to incomplete penetrance of mutations. The remaining mutation that was detected in Families 1 and 2 (c.496G>A) was first reported by Wang et al (13). Our cases were more severely affected (BMI values of patients 1-II-2 and 2-II-1: 42 and 61, respectively) than their case who was a seven-year-old patient with a BMI value of 30.7 kg/m^2^ (13). Other genetic and environmental modifiers would explain differences in the severity of the phenotype of c.496G>A mutation (21).

In summary, the present study provides data regarding MC4R mutations in severe obese children and adolescents from Turkey. We found a higher frequency of MC4R mutations compared to the existing literature.

## Figures and Tables

**Table 1:**
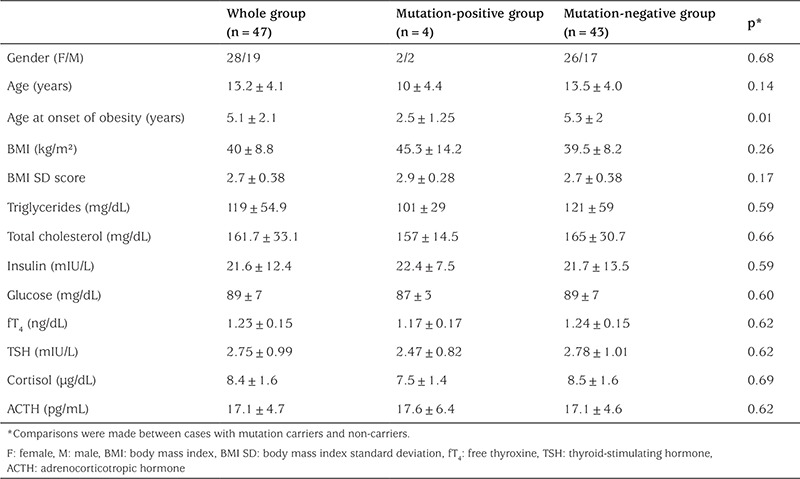
Comparison of anthropometric and biochemical variables between MC4R mutation carriers and non-carriers

**Table 2:**
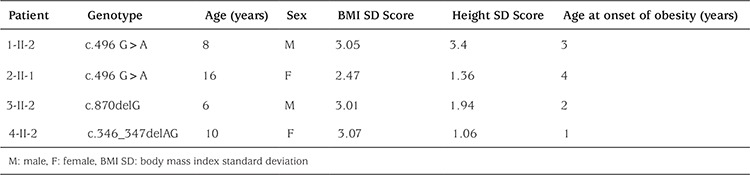
Genotypic and phenotypic characteristics of mutation carriers
